# A study on the intervention of eight-section brocade exercises in combination with comprehensive measures on the physical function status of patients with chronic obstructive pulmonary disease

**DOI:** 10.1016/j.clinsp.2024.100536

**Published:** 2024-12-19

**Authors:** Jiezhen Li, Yan Lei, Meini Li

**Affiliations:** a Comprehensive Department of Traditional Chinese Medicine, The First Affiliated Hospital of Xinjiang Medical University, Xinjiang, PR China; b North District Department of Respiration, Xijing 986 Hospital Department, Fourth Military Medical University, Shaanxi, PR China; c Department of Pulmonary Diseases, Gansu Provincial Hospital of Traditional Chinese Medicine, Gansu, PR China

**Keywords:** Eight-section brocade exercises, Comprehensive intervention, Chronic obstructive pulmonary disease, Function status

## Abstract

**Background:**

Chronic Obstructive Pulmonary Disease (COPD) is a common chronic respiratory disease with a long course and recurrent symptoms, seriously affecting patients' quality of life.

**Objectives:**

This study aimed to explore the interventional value of eight-section brocade exercises in combination with comprehensive measures of the physical function status of patients with COPD.

**Method:**

This is a retrospective study. A total of 94 COPD patients admitted to the studied hospital were divided into two groups according to different intervention methods. The control group was treated with comprehensive intervention, and the research group received eight-section brocade exercises combined with comprehensive intervention.

**Results:**

After an intervention, the research group exhibited longer exercise endurance time (*p* < 0.05); the modified Medical Research Council (mMRC) scores and quality of life scores in the research group were lower than those in the control group (*p* < 0.05); sleep quality scores of the research group being lower than those of the control group (*p* < 0.05); both groups experienced a decrease in adverse emotion scores, with the research group scoring lower than the control group (*p* < 0.05); the research group achieving better physical function status than the control group (*p* < 0.05); the nursing efficiency rate and satisfaction rate in the research group was higher than those in the control group (*p* < 0.05).

**Conclusions:**

Eight-section brocade exercises can enhance the exercise endurance of and improve the lung function of COPD patients, which is of great significance for the recovery of patients’ physical function.

## Highlights

• Eight-section brocade exercises enhance exercise endurance and lung function of COPD patients.
• Eight-section brocade exercises improve the physical function of COPD patients.
• Eight-section brocade exercises improve patient emotions, nursing efficiency, and satisfaction.

## Introduction

Chronic Obstructive Pulmonary Disease (COPD), as a common chronic respiratory disease, has a long course and recurrent symptoms, which seriously affects the quality of life of patients and causes a huge economic burden to the country and society.^1^^,^^2^ Although there are a variety of treatment methods, the rehabilitation methods for the decline of physical function status in COPD patients still need to be further explored and improved. In particular, there are still gaps and deficiencies in the literature on the rehabilitation effect of traditional Chinese fitness Qigong, a Chinese system of physical exercises and breathing control related to tai chi, such as eight-section brocade exercises on COPD patients.^3^ Eight-section brocade exercises are a traditional fitness Qigong that can enhance cardiorespiratory function, muscular strength, and flexibility in the human body through specific movements and breathing methods.^4^, ^5^, ^6^ The combination of comprehensive intervention measures such as diet, psychology, and breathing on the basis of eight-section brocade exercises has a better rehabilitation effect.^7^ However, there are insufficient studies on the effects of the above two interventions on the physical function status of COPD patients.

Therefore, this study aimed to fill this gap and explore the influence of eight-section brocade exercises combined with comprehensive intervention on the physical function status of COPD patients. It was hypothesized that eight-section brocade exercises combined with comprehensive intervention can more effectively improve the exercise endurance, lung function, and overall physical function status of COPD patients than comprehensive intervention alone. In order to verify this hypothesis, 94 COPD patients admitted to the hospital were selected as research subjects and divided into control and research groups. Comprehensive intervention and eight-section brocade exercises combined with comprehensive intervention were implemented respectively, and the effect of intervention was comprehensively explored by evaluating the exercise endurance, lung function, and physical function status of patients. It is hoped that through this study, a more effective rehabilitation method can be provided for COPD patients, helping patients improve their quality of life, reducing the burden of disease, and providing new ideas and directions for research in the field of COPD rehabilitation.

## Materials and methods

### Study design and setting

This is a retrospective study. A total of 94 COPD patients admitted to the hospital were retrospectively selected and divided into two groups according to the different intervention methods.

### Participants

Patients meeting the following inclusion criteria were included: (1) Patients with COPD in a stable period; (2) Patients aged ≤ 80-years; (3) Patients with effective communication ability; (4) Patients meeting the COPD diagnostic criteria (revised in 2021).^8^

Exclusion criteria were as follows: (1) Patients with severe conditions such as severe pneumonia, pulmonary tuberculosis, etc.; (2) Patients with cardiac arrhythmias; (3) Patients with malignant tumors; (4) Patients unable to undergo long-term follow-up.

### Sample size

After screening through the hospital medical record system, the clinical data of 94 patients were included. The control group (*n* = 47) consisted of 25 males and 22 females, with a mean course of disease of (5.08 ± 1.36) years, a mean age of (64.19 ± 3.64) years, 21 smokers, and disease severity grades: 8 cases of grade III, 27 cases of grade II, and 12 cases of grade I. The research group (*n* = 47) consisted of 27 males and 20 females, with a mean course of disease of (5.12 ± 1.29) years, a mean age of (64.37 ± 3.51) years, 19 smokers, and disease severity grades: 7 cases of grade III, 26 cases of grade II, and 14 cases of grade I. The clinical data of the two groups were comparable (*p* > 0.05).

### Interventions

The control group received comprehensive interventions, including (1) Diet: The dietary habits should be adjusted to regulate immunity and resistance in order to more effectively prevent the progression of diseases. Patients should choose a comfortable position, keep the mouth and nasal passages clear, and facilitate the administration of food. (2) Psychology: The nursing staff explained the entire process of operating the ventilator to the patient, ensuring that they had a clear understanding of the entire therapy process and could effectively guide the patient in breathing correctly. (3) Breathing: Simple breathing exercises were performed in a forward-leaning or standing position, and diaphragmatic breathing and pursed-lip breathing were also incorporated.

The research group received a combination of eight-section brocade exercises and comprehensive intervention. The eight-section brocade exercises included the follows: (1) Breathing exercise: The patients were instructed to stand upright, slightly lean forward, naturally place both hands on the sides of the legs, lift the chest and head, inhale, and then exhale in a hunched-back, chest-contracted posture until all the air was completely expelled. This action was repeated for 10 times. (2) Arm swing: The patients' hands hung naturally at their sides, slowly raised their arms above their heads, and then slowly lowered them to their original position, repeating several times. This movement could increase shoulder joint flexibility and improve the coordination of respiratory muscles. (3) Head and tail shake: The patients spread their legs apart and gently shook their heads from side to side, followed by rotating the head left and right. Then, they swayed their waist from side to side, resembling the movement of a tail. This action could help relieve tension in the cervical and lumbar spine and improve spinal flexibility. (4) Abdominal massage with hands: The patients' hands were crossed and placed on their abdomen for massage in a clockwise direction, repeating several times. This motion could promote blood circulation in the abdominal area and enhance the activity of digestive organs. (5) Squat and ball hold: The patients held the ball with both hands, squatted until the thighs were parallel to the ground, and then stood up, repeating this movement several times. This exercise could strengthen the leg muscles and increase lower body strength. (6) Tip-toe raises: The patients stood with their feet together and raised both feet onto their tiptoes simultaneously, repeating this motion several times. This exercise could strengthen the calf muscles and improve foot flexibility. (7) Bend the bow to shoot the tiger: The patients raised both hands overhead and then forcefully pushed them forward, repeating this movement multiple times. This exercise could strengthen the back muscles and increase chest expansion. (8) Closing form: The patients should stand quietly with their feet together, relax their bodies, slowly close their eyes, and use their thoughts to sense every part of their body. This movement could help patients relax both physically and mentally, improving their mental state. The above exercises were conducted under the guidance of nursing staff, with standardized procedures. It was practiced four times a day, with each session lasting 8‒10 min.

### Observation indicator comparisons

#### Exercise endurance

The 6-minute walk test was when a patient walked quickly on a flat surface for 6 min, recording the distance walked. The walking distance was positively correlated with the patient's exercise endurance.

Exercise endurance time refers to the time a patient continued to walk on flat ground until they experienced shortness of breath symptoms.

#### Lung function

The lung function meter (model 8800D) was used to continuously measure the Forced Expiratory Volume in the first second (FEV1), Maximum Voluntary Ventilation (MVV), and Forced Vital Capacity (FVC) for continuous three times. The average of the measurement results was taken as the final value, and the operation was strictly carried out in accordance with the instructions.

#### Dyspnea index (mMRC)

The modified Medical Research Council (mMRC)^9^ scale was used to assess the severity of dyspnea in patients, with 3‒4 points representing very severe, 2 points representing severe, 1 point representing moderate, and 0 points representing mild. The lower score indicates milder symptoms.

#### Quality of life

The St. George's Respiratory Questionnaire (SGRQ)^10^ was used to assess patients' quality of life, consisting of a total of 76 items, primarily covering disease impact, respiratory symptoms, activity limitation, etc. The total score was 100 points, with lower scores indicating better quality of life.

#### Sleep quality

The Pittsburgh Sleep Quality Index (PSQI) was used to assess the sleep quality of patients. The total score was 21 points, with a score of 0‒5 indicating very good sleep quality, a score of 6‒10 indicating fairly good sleep quality, a score of 11‒15 indicating moderate sleep quality, and a score of 16‒21 indicating poor sleep quality.

#### Nursing efficiency

Invalid: Symptoms worsened or showed no improvement; Valid: Symptoms improved; Highly effective: Symptoms disappeared, or most symptoms returned to normal. Efficiencyrate=(Highlyeffective+Valid)/Numberofcases×100%.

#### Satisfaction

The satisfaction of patients with nursing skills, methods, and quality was evaluated using self-designed questionnaires.^11^ Scores below 60 indicate dissatisfied; scores of 60‒80 indicate satisfied, and scores above 80 indicate very satisfied. Satisfaction=(Verysatisfied+Satisfied)/Totalcases×100%.

#### Adverse emotions

The Self-Rating Depression Scale (SDS)^12^ and the Self-Rating Anxiety Scale (SAS)^13^ were used to assess the levels of depression and anxiety, respectively. The total score was 100 points, with 53 points as the cutoff threshold. Scores of 53‒62 indicate mild depression or anxiety; scores of 63‒72 indicate moderate depression or anxiety, and scores greater than 72 indicate severe depression or anxiety.

#### Physical function status

The revised Piper Fatigue Scale^14^ was used to assess the patient's physical function status, including four dimensions: emotional, cognitive, behavioral, and physical fatigue. There was a total of 22 items on the scale, with a total score of 10. A lower score indicates a milder fatigue state.

#### Ethics and endpoint

This study was approved by the ethics committee of Gansu Provincial Hospital of Traditional Chinese Medicine (n° 2023JNB0051). The study followed the STROBE Statement. Due to the retrospective nature of this study, informed consent was waived by the ethics committee of Gansu Provincial Hospital of Traditional Chinese Medicine. The endpoint of this study was the 12th week after the intervention, and all the patients were followed up for improvements in exercise endurance, lung function, dyspnea index, quality of life, sleep quality, nursing efficiency, satisfaction, adverse emotions, and physical function status.

### Statistical methods

The data were analyzed using SPSS 17.0 statistical software. For measurement data, the authors first verified whether it satisfied normal distribution and homogeneity of variance. Under the conditions of normal distribution and homogeneity of variance, if two sets of data were independent, an independent samples *t*-test was used to analyze the mean difference between the two groups (expressed as mean ± standard deviation); If two sets of data were observations of the same group of indicators at different time points or conditions, paired samples *t*-test was used to analyze the differences between paired data. For counting data, the chi square test was used to analyze the proportion differences (%) between categorical variables. All statistical tests were set to have a significance level of *p* < 0.05. When the p-value was less than 0.05, the difference was considered statistically significant.

## Results

### Comparison of exercise endurance between the two groups

Before intervention, no statistically significant differences were observed between the two groups in terms of exercise endurance time and 6 Min Walk Distance (6MWD) (*p* > 0.05); After intervention, both groups showed varying degrees of improvement in endurance, and the research group exhibited longer exercise endurance time and longer 6MWD compared to the control group (*p* < 0.05) (Table 1).

**Table 1:** Comparison of exercise endurance (χ ± *s*).

Group	Number of cases	Exercise endurance time (min)		6-minute walk distance (m)	
		Before intervention	After intervention	Before intervention	After intervention
Control group	47	12.89 ± 2.05	18.42 ± 2.62	246.82 ± 20.82	328.01 ± 24.29
Research group	47	12.86 ± 2.13	23.04 ± 3.72	246.78 ± 19.97	409.58 ± 36.71
*t*	/	0.070	6.961	0.010	12.704
*p*	/	0.945	0.000	0.992	0.000

### Comparison of lung function between the two groups

Before the intervention, there was no statistically significant difference in lung function indicators, including FEV1, MVV, and FVC, between the two groups (*p* > 0.05). After the intervention, both groups showed varying degrees of improvement in lung function, and the research group exhibited higher levels of FEV1, MVV, and FVC compared to the control group (*p* < 0.05) (Table 2).

**Table 2:** Comparison of lung function (χ±*s*).

Group	Number of cases	FEV_1_ (%)		MVV (%)		FVC (%)	
		Before intervention	After intervention	Before intervention	After intervention	Before intervention	After intervention
Control group	47	66.52 ± 5.28	80.36 ± 8.21	53.69 ± 6.21	66.51 ± 7.28	63.18 ± 6.52	75.36 ± 7.08
Research group	47	65.82 ± 6.01	86.19 ± 8.39	53.54 ± 6.07	74.08 ± 8.36	63.21 ± 6.43	82.45 ± 8.04
*t*	/	0.560	3.405	0.118	4.682	0.023	4.537
*p*	/	0.550	0.001	0.906	0.000	0.982	0.000

### Comparison of mMRC and quality of life between the two groups

Before intervention, no statistically significant differences were observed in terms of mMRC and quality of life between the two groups (*p* > 0.05); After intervention, both groups showed varying degrees of improvement in mMRC and quality of life, and the research group showed lower mMRC scores and quality of life scores compared to the control group (*p* < 0.05) (Table 3).

**Table 3:** Comparison of mMRC and quality of life (χ ± *s*).

Group	Number of cases	mMRC		SGRQ score (points)	
		Before intervention	After intervention	Before intervention	After intervention
Control group	47	2.58 ± 0.72	1.75 ± 0.58	49.52 ± 7.05	42.82 ± 4.28
Research group	47	2.56 ± 0.74	1.31 ± 0.41	49.25 ± 7.25	35.67 ± 4.04
*t*	/	0.133	4.267	0.183	8.329
*p*	/	0.895	0.000	0.855	0.000

### Comparison of sleep quality between the two groups

Before intervention, there was no statistically significant difference in sleep quality scores between the two groups (*p* > 0.05); After intervention, the sleep quality scores of both groups decreased, with the sleep quality scores of the research group being lower than those of the control group (*p* < 0.05) (Table 4).

**Table 4:** Comparison of sleep quality between the two groups (χ ± *s*).

Group	Number of cases	mMRC	
		Before intervention	After intervention
Control group	47	9.27 ± 1.26	7.02 ± 1.05
Research group	47	9.24 ± 1.34	4.36 ± 0.78
*t*	/	0.112	13.942
*p*	/	0.911	0.000

### Comparison of nursing efficiency between the two groups

**Fig. 1:**
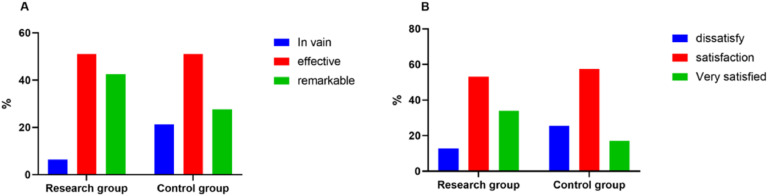
Nursing efficiency.

The research group achieved a nursing efficiency rate of 93.62% (44/47) and a satisfaction rate of 87.23% (41/47), which was higher than the nursing efficiency rate of 78.72% (37/47) and satisfaction rate of 74.47% (35/47) of the control group (*p* < 0.05) (Fig. 1).

### Comparison of adverse emotions between the two groups

**Fig. 2:**
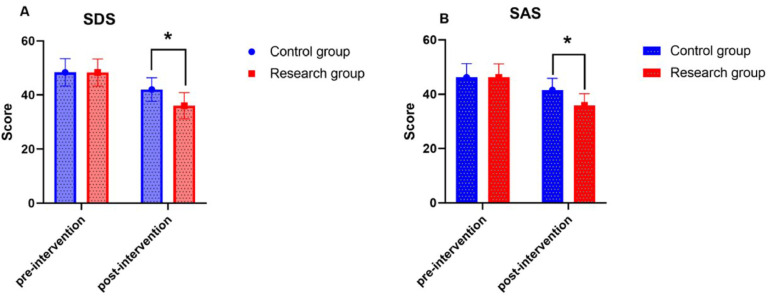
Adverse emotions.

Before intervention, there was no statistically significant difference in the SDS and SAS scores between the two groups (*p* > 0.05); After intervention, both groups experienced a decrease in adverse emotion scores, with the research group scoring lower than the control group (*p* < 0.05) (Fig. 2).

### Comparison of physical function status between the two groups

**Fig. 3:**
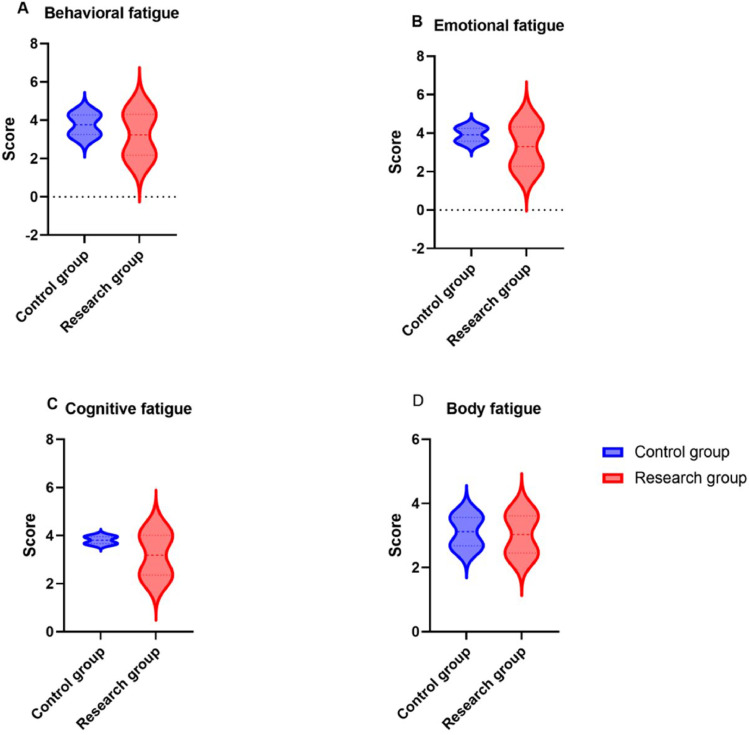
Physical function status.

Before intervention, there was no statistically significant difference in the physical function status between the two groups (*p* > 0.05); After intervention, the scores for physical function status in both groups decreased, with the research group achieving better physical function status than the control group (*p* < 0.05) (Fig. 3).

## Discussion

Patients with COPD often experience a significant decline in lung function, and lung function tests are an objective method for assessing their pulmonary function.^15^, ^16^, ^17^, ^18^ The results of this study showed that, after the intervention, the research group exhibited higher lung function indicators, including FEV1, MVV, and FVC, compared to the control group (*p* < 0.05). From the perspective of traditional Chinese medicine, respiratory training helps promote the generation of vital energy and blood, which has a tonifying effect on the spleen.^19^ From a modern perspective, it can enhance the endurance of the diaphragm muscles in patients and promote improved respiratory efficiency.^20^ Breathing exercises relax the diaphragm muscle, increasing the tidal volume of respiration, and effectively facilitating pulmonary gas exchange.^21^ Eight-section brocade inhalation training can reduce the contraction of the diaphragm muscles, increase chest cavity volume with the combined action of auxiliary muscles and the diaphragm muscles, further increase lung inhalation capacity, and restore normal respiratory function.^22^ Eight-section brocade exercises integrate mind, body, and breath, emphasizing willpower training, which distinguishes it from other rehabilitation methods. COPD patients may experience varying degrees of clinical symptoms such as dyspnea and cough, leading to reduced physical activity and shortened exercise duration.^23^ The 6MWD can be used to assess changes in respiratory symptoms in patients. In the present study, after the intervention, patients in the research group showed longer exercise endurance time and longer 6MWD compared to the control group, and the physical function status was better than that of the control group. The results confirm that eight-section brocade exercises are a low-intensity and regular exercise method that helps enhance patients' cardiopulmonary function, improves the circulation of vital energy and blood, and contributes to increasing vascular density and elasticity in patients, thereby further reducing the burden on their hearts.

COPD patients often experience a decline in physical function and a reduced quality of life. Therefore, it is of great significance to intervene in the physical function status of COPD patients.^24^ In this study, the quality of life and sleep quality of the patients improved to varying degrees after a combination of eight-section brocade exercises and comprehensive intervention. The primary reason for this improvement is that eight-section brocade exercises can simultaneously coordinate the patient's mind and body. Through regular breathing, eight-section brocade exercises enhance lung function, promote metabolism, and increase oxygen intake and ventilation, which boost the patient's energy levels and facilitate the recovery of their condition. Eight-section brocade exercises are a training approach based on human physiology and kinematics characteristics, which combine both static and dynamic exercises, allowing patients to achieve the benefits of unblocking meridians and promoting cardiovascular health during the movement. Patients experience both physical and mental conditioning, accompanied by a sense of relaxation. Their emotional state also undergoes changes, leading to a significant improvement in anxiety and depression.

The advantage of this study is that eight-section brocade exercises, a rehabilitation method with Chinese characteristics, combined with comprehensive intervention methods, provide a new idea for the rehabilitation treatment of COPD patients. However, this study also has some limitations. First, the sample size was relatively small, which may have affected the generalizability of the results. Second, the study only observed the short-term effects after intervention, and there was a lack of long-term follow-up data to assess the persistence of treatment effects. Finally, this study only included eight-section brocade exercises in conjunction with comprehensive intervention and did not compare with other intervention methods, so whether eight-section brocade exercises combined with comprehensive intervention is the optimal COPD rehabilitation program still needs to be validated in further research.

Because this study did not include long-term follow-up, it is not possible to accurately assess the durability of treatment effects and long-term safety. In addition, the subjects in this study may have individual differences, such as age, gender, disease course, and other factors that may have an impact on the study results, but these factors were not fully controlled and considered in this study.

## Conclusion

In conclusion, eight-section brocade exercises combined with comprehensive intervention can enhance the exercise endurance of and improve the lung function of COPD patients, which is of great significance for the recovery of patient's physical function and improvement of their quality of life. Future studies should further expand the sample size and increase long-term follow-up observation to verify the stability and durability of the results of this study. At the same time, a multi-center, large-sample randomized controlled trial should be conducted to evaluate the optimality and safety of eight-section brocade exercises combined with comprehensive intervention in the rehabilitation treatment of COPD. In addition, the combination of eight-section brocade exercises and other rehabilitation methods can be explored to find a more comprehensive and effective COPD rehabilitation treatment program.

## Author's contribution

All authors contributed to the study conception and design, material preparation, data collection, and analysis. The first draft of the manuscript was written by Jiezhen Li and Yan Lei. Meini Li commented on previous versions of the manuscript. All authors read and approved the final manuscript.

## Funding

This research did not receive any specific grant from funding agencies in the public, commercial, or not-for-profit sectors.

## Declaration of competing interest

The authors declare no conflicts of interest.
